# Institutional review board challenges related to community-based participatory research on human exposure to environmental toxins: A case study

**DOI:** 10.1186/1476-069X-9-39

**Published:** 2010-07-16

**Authors:** Phil Brown, Rachel Morello-Frosch, J G Brody, Rebecca Gasior Altman, Ruthann A Rudel, Laura Senier, Carla Pérez, Ruth Simpson

**Affiliations:** 1 Brown University, Department of Sociology and Center for Environmental Studies, Box 1916, Providence RI 02912, USA; 2 University of California, Berkeley School of Public Health and Department of Environmental Science, Policy and Management, 137 Mulford Hall, Berkeley CA 94720-3114, USA; 3 Silent Spring Institute, 29 Crafts Street, Newton MA 02458, USA; 4 Tufts University Department of Community Health, 112 Packard Ave., Medford MA 02555, USA; 5 University of Wisconsin Department of Family Medicine and Department of Rural Sociology, 1450 Linden Drive Madison, WI 53706-1522, USA; 6 Communities for a Better Environment, 1904 Franklin Street, Suite 600, Oakland CA 94612, USA; 7 Bryn Mawr College, Department of Sociology, 101 North Merion Avenue Bryn Mawr PA 19010-2899, USA

## Abstract

**Background:**

We report on the challenges of obtaining Institutional Review Board (IRB) coverage for a community-based participatory research (CBPR) environmental justice project, which involved reporting biomonitoring and household exposure results to participants, and included lay participation in research.

**Methods:**

We draw on our experiences guiding a multi-partner CBPR project through university and state Institutional Review Board reviews, and other CBPR colleagues' written accounts and conference presentations and discussions. We also interviewed academics involved in CBPR to learn of their challenges with Institutional Review Boards.

**Results:**

We found that Institutional Review Boards are generally unfamiliar with CBPR, reluctant to oversee community partners, and resistant to ongoing researcher-participant interaction. Institutional Review Boards sometimes unintentionally violate the very principles of beneficence and justice which they are supposed to uphold. For example, some Institutional Review Boards refuse to allow report-back of individual data to participants, which contradicts the CBPR principles that guide a growing number of projects. This causes significant delays and may divert research and dissemination efforts. Our extensive education of our university Institutional Review Board convinced them to provide human subjects protection coverage for two community-based organizations in our partnership.

**Conclusions:**

IRBs and funders should develop clear, routine review guidelines that respect the unique qualities of CBPR, while researchers and community partners can educate IRB staff and board members about the objectives, ethical frameworks, and research methods of CBPR. These strategies can better protect research participants from the harm of unnecessary delays and exclusion from the research process, while facilitating the ethical communication of study results to participants and communities.

## Background

In 1979 the Belmont Report established principles for the use of human subjects in scientific research. Developed partly in response to the Tuskegee syphilis study, Belmont identified three basic principles governing the ethical use of human research subjects. The first of these, "respect for persons," stressed that an individual's decision to become a research participant must be voluntary, and called for special protection for those who lacked the capacity to make such a decision themselves. The second principle, "beneficence," called on researchers to "do no harm" or barring that, to maximize the benefits of their research while reducing as much as possible the risk to the subject. Finally, the principle of "justice" required careful attention to the fair distribution of risks and benefits, calling on researchers to select subjects only "for reasons directly related to the problem being studied" and to vigilantly avoid the selection of subjects for "their easy availability, their compromised position, or their manipulability." Justice also required that those who bear the risks of research should, whenever possible, be among the first to benefit from its insights [1].

Implementation of the Belmont Report principles fell to institutional review boards (IRBs) that protect individual research participants through confidentiality, informed consent, and oversight. But while IRBs have been the traditional enforcers of the Belmont principles, they are not the only place where those principles have found expression. Those same principles form the basis of community-based participatory research (CBPR), a method that has become increasingly important in the work of environmental justice and environmental public health activists who are engaging more directly in scientific research design and the collection and analysis of individual-level human data [2-5]. CBPR explicitly focuses on problems that affect whole communities--environmental toxins, for example--and thus is different from most biomedical research which takes the individual as its primary subject. In CBPR projects, researchers work closely with community members and community-based organizations to develop appropriate research agendas, conduct analyses, and disseminate results and information. This merging of community interests and community action reflects another distinct quality of CBPR: its commitment to advocacy for the public good and creating open access to information.

CBPR takes "respect for persons" to a new level: not only do study participants voluntarily participate in research, they *actively *participate in research design, data collection, analysis, implementation, and dissemination. This inclusion reflects CBPR's commitment to the principles of "beneficence" and "justice," as the active involvement and scrutiny of study participants encourages the fair assessment and distribution of the research's risks and benefits. Further, the practice of giving research participants the decision to have full access to research results helps ensure they have sufficient information to make informed choices during and after the study, and is thus consistent with Belmont's emphasis on informed consent. In the process, research "subjects" are transformed into research "participants."

Ironically, however, *IRB review of CBPR projects can result in unintended violations of the very principles they seek to uphold*. This is due in part to implicit assumptions embedded in the Belmont Report that were adopted by IRBs but that contradict other CBPR principles. For example, IRBs, following Belmont, assume that the research participant is an *individual*, whereas CBPR sees research participants as both individuals and as a *community *of individuals [6]. This difference has profound implications for confidentiality, the dissemination of information, and the assessment of risks and benefits. Similarly, CBPR's inclusion of laypersons and others traditionally outside the *research *process means university IRBs must consider people who are traditionally outside their *review *process (such as community-based organizations with no formal relationship to the university), which can lead to misunderstandings and unnecessary delays as IRBs deliberate whether and how to extend their jurisdiction into new territory. These differences and the general unfamiliarity of many IRBs that we sampled with the CBPR approach can result in undue obstacles and extensive delays, and hence can reduce the benefit of the research for the research participants and even cause them harm as they anxiously wait unnecessarily long to receive results that may directly affect their own well-being and that of their families and their communities, or are "protected" from information that could inform their own decisions about their future health and welfare. A third example arises when communities request that studies be conducted, and work with scientific collaborators to design and fund the project, but when IRBs either delay, or deny, oversight, hindering communities from investigating environmental problems in their communities.

The increasing use of CBPR in the work of environmental justice and environmental public health activists, scholars, and practitioners makes it particularly important that researchers, funders, community members, and university administrators work towards defining IRB oversight procedures that will advance CBPR projects instead of inadvertently hindering them. We draw on our own experiences to highlight problems that arise when Belmont principles meet the IRB review process, and how those problems undermine rather than support the spirit of the Belmont Report. We supplement those accounts with reports from other CBPR researchers, and propose procedures that will allow IRBs and CBPR researchers to work together in a review process that promotes their shared goal of ethical, principled, and beneficial research.

## Methods

We draw our data from our own experiences with the IRB process and from the experiences of other researchers using CBPR to examine issues of environmental justice. Our research collaborative involves a partnership between four institutions: Brown University, Silent Spring Institute (a non-profit environmental health research organization based in Massachusetts), the University of California, Berkeley, and Communities for a Better Environment (CBE; a California environmental justice organization that combines organizing, advocacy, litigation, and research). Our project entails sampling of household air and dust in three study sites: Richmond and Bolinas in California, and Cape Cod in Massachusetts. We also conducted human biomonitoring on Cape Cod. In response to community concerns about environmental justice and about environmental links to breast cancer, we tested for endocrine-disrupting compounds and for additional pollutants from industry and transportation corridors near the Richmond site. The project reports individual results to study participants who choose to receive them, and aggregate results through community meetings, peer-reviewed publications, and media outreach. Brown University's IRB was initially reluctant to oversee the researchers in the community partner organizations, but ultimately facilitated an effective human subjects protection oversight strategy for the entire collaborative. Hence, our experience serves as a model for others, and adds weight to a changing research protection paradigm that understands and values community-based participatory research.

We also incorporate consultations with twelve other colleagues collaborating on CBPR projects at other institutions. We selected these colleagues from the list of current projects funded by the National Institute of Environmental Health Sciences (NIEHS) Environmental Justice Program. Because we were, at the time, only seeking information to guide our own IRB request, we were not subject to human subjects protection oversight for these consultations. Therefore, we report on lessons learned without direct reference to the researchers or their institutions. While preparing for a research proposal to study these IRB issues in greater depth, we spoke with four additional researchers involved in biomonitoring and household exposure studies.

We also draw data from participants at a workshop we led at the NIEHS Environmental Justice Program grantees conference in Talkeetna, Alaska [7] in September 2005, and our own participation in a workshop on community review boards led by West Harlem Environmental Action (WEACT) at the NIEHS Environmental Justice Program grantees conference in Boston in December 2007. Uncited material stems from our interviews, conversations, and observations.

## Results

In their frustration with IRBs, CBPR researchers reported to us that they have joined a growing group of scholars concerned that the review process has become too formulaic and inflexible. Designed around biomedical and behavioral research, IRB review is often inappropriate to the methods, challenges and objectives of other approaches to research from across the disciplines. Social scientists, for example, have criticized the application of stringent informed consent procedures for low risk, non-intrusive interview research, such as interviewing public officials, who are legally obligated to reply to citizen queries [8].

CBPR researchers told us they face a unique set of challenges in the IRB process. The differing assumptions that CBPR researchers and IRBs bring to the process come into sharpest relief regarding IRB's opposition to two particular CBPR practices: (1) layperson participation in the research process and (2) the report-back of individual results to study participants. Laypeople and community organizations who are involved in CBPR research are outside the conventional jurisdiction of institutional IRBs. Meanwhile, the CBPR practice of report-back, and especially the philosophy of openness that informs it, challenges IRB assumptions about who controls the flow of data produced in human subjects research, when and whether those data should be made available to members of an affected community, and what the nature and duration of the researcher-subject relationship should be.

In recent practice, some traditional clinical research has led to inclusion of community advisory boards and to explicit assumptions about the need to consider broad community benefits. For example, some AIDS research and breast cancer research has involved community members in central ways [7]. Indeed, this may have helped some IRBs understand the need to transcend traditional models for human subjects review. In this light, the distinctions between traditional research and CBPR may seem to be more blurred. Still, our experience, combined with our familiarity with many CBPR projects, leads us to believe that CBPR represents a major shift in research and engagement with study communities that makes it qualitatively different, even from the occasional community-oriented AIDS and breast cancer studies that exist under the umbrella of clinical research. These issues are highlighted and discussed below.

### Institutional review board oversight of community-based participatory research

CBPR researchers reported being informed by a major shift in research ethics. Unlike conventional research in which the researcher's relationship with the subject is unilateral and unidirectional, CBPR is collaborative, inviting community members and community-based organizations to work alongside the scientists, social scientists, and medical professionals studying that community. Laypersons help define the research agenda, form research questions, carry out the study, and disseminate results and information back to the community and other relevant parties, such as public health practitioners and social service agencies [5,9]. CBPR thus enters a "post-Belmont era", blurring the traditional roles of researcher and subject and taking seriously the insight, energy, and objectives that members of the affected community bring.

The lay involvement is one way CBPR expresses the Belmont principle of "respect for persons," intended to protect research participants from being objectified and dehumanized. By encouraging the active involvement of research participants in the research process, CBPR greatly reduces the chances that they will be objectified in the first place. As participants in the design and implementation of the research plan, members of the affected community have a level of "informed consent" far deeper than typically occurs in conventional research. Thus CBPR achieves "respect for persons" by democratizing the research process and encouraging scientific and medical experts to work alongside laypersons, rather than treating them as objects of study.

Facilitating collaboration and communication among scientists, medical professionals, and laypersons has its own challenges, but often the biggest hurdle CBPR researchers discussed was how to introduce IRBs to the idea. In our and our colleagues' experiences, IRBs are uneasy when community-based organizations (CBOs) serve as formal partners in a research initiative. The main problem is jurisdictional: university IRBs are reluctant to oversee human subjects protection compliance for partner organizations outside the university. That discomfort is sometimes magnified by the routine activities of the CBO. For example, a CBO's process of seeking feedback from its constituency--evaluating whether a conference had successfully reached its target audience, for example--could appear to an academic IRB as human subjects research requiring review. This could appear to the CBO as an unwelcome intrusion into its internal affairs (exceptions for such routine activities already exist in federal regulations and could be a basis for exempting CBO educational evaluations, although IRBs are not always aware of this). IRBs may be particularly disturbed when a CBO challenges traditional academic norms by engaging in both research and advocacy, leading IRBs to attempt to influence activities that many CBOs believe should be under their own control.

Many of these problems were apparent when we sought human subjects review from Brown University's IRB for a project that involved two CBO partners: Silent Spring Institute and Communities for a Better Environment. Silent Spring Institute was the principal investigator for two reasons. First, Silent Spring Institute originated the study of large numbers of diverse analytes in homes, and are the foremost researchers in this exposure assessment field; as a result, are the only partners on this team with the established capacity to design and implement its core exposure assessment science, and originated the idea of studying participants' report-back experiences. Second, NIEHS had encouraged CBPR project teams to promote leadership by CBO partners. The IRB was initially reluctant to oversee human subjects protection for researchers outside the university, doubting its own capacity to oversee and hold accountable the research work of independent organizations that were not legal entities of the university. But in fact, this and other IRBs routinely oversee research conducted in other countries by locally hired researchers. Of primary concern was the possibility that some CBO activities might violate federal standards and jeopardize federally-funded research activities and the reputation of the university as a whole.

From our standpoint, their concerns about jurisdiction were unfounded. The Department of Health and Human Services' Policy for the Protection of Human Subjects makes clear that CBOs can be reprimanded directly by the HHS Office for Human Research Protection (OHRP) for a violation of federal regulations for the protection of human subjects [10]. Brown's IRB could have contacted the OHRP directly with any concern about a CBO partner's ability to conduct research or the CBO's ability to ensure the protection of human subjects in federally funded research. All CBOs conducting research are required to obtain and periodically update an OHRP assurance of compliance with human subjects protection guidelines and must report any suspension or termination of research by an IRB.

To allay IRB concerns, we demonstrated that our community partners were experienced in scientific research and well-versed in human subjects protection protocols. Silent Spring Institute has a long track record of obtaining state IRB approval for environmental health research supported by state, federal, and private funds. Communities for a Better Environment (CBE) is a long-established organization whose research using secondary data (such as oil refinery emissions, for example) was well-known to academic institutions and government agencies. Moreover, individual CBE staff members had previous experience working for universities and health providers where they had participated in human subjects research. Further, staff from both Silent Spring and CBE completed human subjects protection training through the National Institute of Health's online certification training and exam.

Despite these credentials, the Brown University IRB remained reluctant. Deciding NIH certification was insufficient, it required that staff from both organizations also take the Collaborative Institutional Training Initiative (CITI) online course and exam, even though it does not address the human subjects issues unique to CBPR projects. In addition, the training module assumed familiarity with online electronic systems, comfort with multiple-choice test-taking skills, and strong English literacy-all potentially disruptive to an environmental justice project, for example, if staffed by people who speak English as a second language or have little or no experience navigating web-based programs. The five or six hours required to complete the training added to the frustration of already over-extended CBOs. At the NIEHS Environmental Justice grantees conferences and other events, our colleagues described similar problems, revealing that we were not alone in this experience.

Ideally we would have turned to NIEHS for help getting community partners through our university IRB process. Unfortunately, although NIEHS promotes CBPR research, it does not offer guidance to IRBs for reviewing academic-community partnerships. The lack of guidance from NIEHS meant that both researchers and IRB staff members were on their own to resolve unique and sometimes conflicting institutional concerns. For example, we developed procedures making Brown University faculty partners responsible for protecting study participants in interviews, human and household sample collection, and record-keeping activities. Faculty partners agreed to make quarterly visits to Communities for a Better Environment and Silent Spring to check record-keeping and data storage protocols, and reported to the IRB on the collaborative's adherence to approved study protocols.

In the end, the IRB agreed to provide oversight for our research collaborative, but at first approved oversight for only eighteen months of a four-year project. After continued dialogue and negotiation, the IRB ultimately agreed to continue for the full duration of the project. While we were pleased to have obtained approval, the many problems in the process caused substantial delays before the project could begin and also during critical phases of the project as it progressed.

### Who gets to know? Conflicts over dissemination of study results

As we learned from interviews, IRBs frequently clash with CBPR researchers over their practices for the dissemination of information and results. IRBs are accustomed to overseeing conventional research projects where study participants often are not informed of personal results that lack regulatory or clinical significance. The CBPR practice of having study participants work alongside researchers confounds assumptions about who should control and have access to the resulting data.

But in CBPR, the participants' right to have access to the results of research derives from a source more fundamental than their own participation in the research effort. CBPR posits that primary ownership of collected data logically lies with the participants from whom the original samples were taken [11]. From the CBPR perspective, it would be inappropriate and unjust to deny full access to information that came from their own bodies, homes, and communities, and that owes its existence to their willing participation.

Researchers told us that IRBs frequently express concerns that disseminating uncertain data may harm human subjects. For example, the participant might be psychologically harmed by receiving results if their clinical significance is unknown or if no valid options exist to address the potential health risks they reveal [12]. Indeed, the National Bioethics Advisory Committee [13] issued guidelines directing researchers to report biomarkers only when health implications are significant and recourse is available. These discussions may not, however, have adequately considered the distinction between communications related to genetic biomarkers, which are not modifiable, and chemical exposures, many of which are modifiable. When these clinical guidelines are applied to chemical exposure research, people are left unaware of the presence in their own bodies of foreign substances known to be harmful in animal studies, and sometimes in *in vitro *and human studies. From the perspective of CBPR, such guidelines are an affront to individuals' and communities' right-to-know, and by extension tarnish the scientific process.

In fact, IRBs' concern with confidentiality may violate federal regulations, which state that "*When appropriate*, there are adequate provisions to protect the privacy of subjects and to maintain the confidentiality of data (emphasis added)." (45 CFR 46.111(a)(7)). This phrase, "when appropriate," is highly germane to CBPR, and IRBs have flexibility in determining when confidentiality is appropriate. If people want their personal data made public, there is no appropriate reason to restrict the release of that information.

In CBPR, "respect for persons" requires and reinforces a commitment to "report-back," an ethical practice that encourages as much as possible the dissemination of individual and aggregate-level results to those study participants who want to have them. This openness is valued because it democratizes knowledge production and helps restructure unequal power relationships [14], especially disparities in access to knowledge that traditionally characterize lay-professional relationships [15]. CBPR also assumes that the sharing of knowledge between researchers and participants empowers communities and individuals to use scientific evidence to advocate for their own interests [11]. Report-back thus upholds the principle of beneficence by giving participants access to information crucial for their health and well-being, and by empowering them to act [2]. From a beneficence perspective, even information about an exposure for which a corresponding risk relationship is not available can have some benefits to participants, such as enabling personal exposure reduction. Through the sharing of aggregate-level data, report-back can also make communities aware of a problem in their midst, and give them both the motivation and the evidence they need to organize community members into collective action. Our project has spent considerable effort reporting data to individual participants and to community gatherings, in order to advance general knowledge, build local capacity for community improvement, and help policy shifts in government and corporate practice [16,17].

As we and our colleagues discovered, sometimes the voluntary sharing of information in CBPR was foreign to IRB conceptions of confidentiality. For example, in the "Body Burden" study (a joint project of Environmental Working Group, Mt. Sinai School of Medicine, and Commonweal [18]) researchers discovered 167 pollutants (out of the 211 tested) in the blood and urine of nine volunteers, with an average of 56 carcinogens in each person. Aggregate study results appeared in *Public Health Reports *[19], but study participants voluntarily placed their individual data on the internet, with photos and personal biographies to accompany the contaminant data [18]. Voluntary though it was, the website would not have sat well with most IRBs, who would perceive that as a violation of confidentiality.

Similarly, we learned from colleagues that IRBs were frequently bothered by the interactive researcher-participant exchange typical of CBPR, one which can change as the project develops. For example, community residents' requests for information to reduce exposure, and our own sense of responsibility, led us to build into our project an intervention phase for reducing household toxics use. We also distributed information sheets to participants about non-toxic alternatives and environmental organizations. We saw our actions as akin to healthcare workers providing treatment and prevention advice in medical screening.

The changing relationship between researcher and study participant is crucial to CBPR but can be troublesome to IRBs accustomed to a more traditional researcher-subject relationship. For example, some researchers noted that academic IRBs require "passive" individual report-back protocols that restrict researchers from proactively asking participants if they want to receive and discuss results. Instead, researchers may only inform study participants how they can contact researchers should they wish to receive individual results. The organic and dynamic nature of researcher-participant interaction in CBPR may be problematic for a review process that assumes all researcher-subject interactions will be planned and obliges the review board to oversee all such exchanges. IRBs may feel obliged to review each phase of that relationship and any and all communications between researcher and participant before the research can proceed. This causes delays for CBPR projects that can delay the research, prevent study participants from getting information necessary for their health and well-being, and may threaten the development of a successful academic-community collaboration.

Such delays and disruptions occurred in the Cape Cod project when we sought to report data to participants. Because many study participants were initially identified through the Massachusetts Cancer Registry, the Department of Public Health's Research and Data Access Review (RaDAR) Committee had jurisdiction over the project. As a result, any later stages of work with these participants (such as developing protocols to inform them of their individual household sampling and biomonitoring results) had to be cleared by the RaDAR Committee. Unfortunately, that review process took many months, and significant difficulties arose with how the RaDAR Committee and others within the Massachusetts Department of Public Health (DPH) approached our CBPR project. One delay occurred when Cape Cod homes with high levels of chemicals were retested to get more detailed measurements that could potentially determine sources of contamination and point to possible remediation strategies. The DPH Bureau of Environmental Health Assessment initially asked to review each letter that went to every one of these households, raising the possibility of protracted negotiation about the scientific interpretation of each individual's finding. Finally, after a meeting with Silent Spring Institute and Brown University researchers, they approved a prototype letter that would be tailored by the research team for each individual's results.

The DPH action was an example of IRB review threatening the very principles it hopes to uphold--in this case the principles of beneficence and justice. Recurring delays from lengthy reviews, on top of funding cuts, kept study participants from getting their results in a timely fashion and jeopardized relations between the research team and participants, thus threatening good CBPR practice. Women who had provided human tissue and household dust and air samples for the study were understandably disconcerted with the delay in receiving their results. Fortunately, Silent Spring Institute's respected status in the community and its skillful handling of the delay enabled participants to stay informed on the progress of the research.

IRB reluctance concerning ongoing researcher-subject interaction was also manifest in its resistance to storing samples in this study. Exposure assessment research is a rapidly evolving field where new analytical methods and knowledge about chemicals in consumer products are constantly improving. Stored samples provide an opportunity to re-analyze data as new research methods become available and more cost-effective. For example, a major breast cancer study published in 2007 used stored samples to show the effects of early exposure to DDT. The study reanalyzed blood samples that had been donated between 1959 and 1967 for a child health and development study. The results showed that women exposed to the highest levels of DDT before mid-adolescence had a five-fold increase in breast cancer risk [20]. As many women heavily exposed to DDT in childhood are still under fifty, we may see startling effects in the coming decades. Had those samples been destroyed, the loss to public health would have been monumental.

In one instance during the Cape Cod project, the DPH threatened to require the destruction of environmental and biological samples immediately after the first laboratory chemical analyses were completed, even though study participants had even given informed consent to allow their household air, dust, and tissue data to be retained for ten years for further analysis. The DPH requirement would have undermined one of the critical goals of the research project - to identify sources of endocrine-disrupting compounds in homes; new assays might be developed in the future to detect additional EDCs, and if samples were destroyed, researchers would miss the opportunity to analyze them. After much negotiation, the RaDAR Committee required the research team to get new consent from study participants in order to continue storing their samples at the research laboratory.

The Cape Cod case demonstrated how maintaining such samples can become crucial for reasons unforeseeable at the time of IRB review. During the research, the study team unexpectedly found breakdown products of a banned flame retardant. Had the samples been destroyed, researchers would have been unable to retest the samples to confirm the parent flame-retardant as the source of the residues. When research participants want their own samples destroyed, their wishes should, of course, be respected, but in the absence of those wishes, IRB requirements to destroy biological specimens can in fact contravene the Belmont principles to maximize research benefits for study participants. The last few years have seen a dramatic increase in knowledge about flame retardants, especially PBDEs, making these samples retroactively part of the front line of public health [21-23].

Despite significant obstacles and delays related to IRB review, our collaborative moved the Massachusetts RaDAR Committee on several critical issues, through continual pressure by academic partners, Silent Spring Institute, supporters from the Massachusetts Breast Cancer Coalition, pro bono legal representation, and state legislators. The Brown University IRB found the experience valuable, too, and they invited us to give a presentation at a statewide research conference they organized in 2007.

### Educating the institutional review board about community-based participatory research

While attempting to shepherd CBPR projects through the IRB process, we and our colleagues at other institutions employed a range of strategies with varying degrees of success. For example, to help the Cape Cod project through Brown University's IRB process, we prepared extensive memos to our university IRB that laid out the history and practices of CBPR, bolstered by extensive in-person dialogue and email with IRB staff. We demonstrated precedent by showing that other researchers at prominent institutions had successfully carried out this kind of collaborative work while observing sound ethical practices, and that another institutional review board had approved such multi-partner collaborative research.

One collaborative researcher we consulted suggested researching IRB members in order to assess their familiarity with CBPR. At one western state university, one faculty member invited the IRB and human subjects administration to an all-day CBPR workshop to improve overall understanding of the principles of CBPR and to establish regular communication between researchers and IRBs. Despite the many real and potential obstacles to collaborative projects, some academic IRBs understand the unique circumstances inherent in CBPR work, and go out of their way to facilitate human subjects protection oversight. Still, in our consultations with other CBPR initiatives, we learned of only one case other than ours where the IRB of a major research university agreed to be the IRB of record for a CBO partner that was a principal investigator in a community-academic research collaborative.

## Discussion

Research collaborations in the CBPR arena--and especially those dealing with environmental justice--have encountered obstacles from both university and governmental IRBs. Unnecessary roadblocks have caused costly delays for CBPR projects and have led project partners to worry that residents in affected communities might lose faith in the researchers and the scientific enterprise itself. With the aid of colleagues in other partnerships, we were able to amass supportive evidence to make our case for our university IRB to cover all three community and academic partners in our project, but only after extensive negotiation and pressure from the Massachusetts Breast Cancer Coalition, and other constituents.

The problems that arise in IRB review of CBPR projects stem from the different assumptions and objectives of diverse parties. An effective solution requires effort by all involved: IRBs, CBPR researchers, CBO partners, and funding agencies. We suggest the following guiding principles for successfully navigating multi-partner CBPR projects through the IRB process (Appendix 1).

### What community-based participatory researchers and their community partners can do

#### 1) Take time to educate the IRB

We found that helping IRBs learn about the objectives and methods of CBPR proved useful in the review process. IRB members may be unfamiliar with CBPR and might benefit from presentations on the history of the work, its basic principles, the funding agencies that support it, the scientific and community benefits of CBPR, and the unique ethical considerations it raises. Getting to know the IRB members in advance of the review process can help researchers assess the extent of education about CBPR that may be necessary.

#### 2) Make sure academic IRBs know community partners

Academic researchers should try to connect community partners with IRB staff to demonstrate the community's involvement in the research process and how their perspective on human subjects protection is key to the project's success. This might include inviting community partners to meetings with the IRB. Research partners can include this "community consent" in their IRB application. If the IRB lacks the familiarity, experience, or the skill set necessary for assessing the ethical issues posed by a research project, an outside expert should be brought in to educate the board [24].

### What institutional review boards and funding institutions can do

#### 1) Keep abreast of CBPR and other cutting-edge research approaches

Just as IRB officials keep up with the literature on conventional human subjects research, they need to keep current on the CBPR literature. Rather than waiting until they are approached for approval of a CBPR project, IRBs should prepare themselves in advance for that encounter. Attending meetings and conferences where CBPR is broadly discussed (e.g. Community-Campus Partnerships for Health, International Society for Environmental Epidemiology, International Society for Exposure Analysis, National Institute of Environmental Health Sciences conferences of their Partnerships for Environmental Public Health and other conferences) or inviting CBPR experts to share their experiences would help IRBs better understand CBPR approaches.

#### 2) Develop routine procedures for the review of CBPR projects

Routinizing IRB review of CBPR would keep new applicants from having to reinvent the wheel and would increase the viability of CBPR projects. The procedure could be developed incrementally as successful projects provide models for others. Alternatively, federal funding agencies could proactively develop guidelines. For example, NIEHS could contract out the development of a protocol to a research institution that deals with ongoing CBPR issues (such as Campus-Community Partnerships for Health, a nonprofit organization that has created partnerships between 1,500 communities and campuses). The resulting guidelines could be posted on relevant websites and form the basis for training sessions.

If IRBs are unfamiliar with CBPR, then they are not conducting their review according to federal regulations, which requires IRBs to be sufficiently qualified through the experience and qualifications of its members, including qualifications and awareness of community attitudes (45 CFR 46.107 (a)). Hence, IRBs are not meeting official requirements if they are unfamiliar with CBPR processes and lack members and advisors with such competence.

#### 3) Provide clear guidance and tools for navigating IRB issues unique to academic-community collaboratives

Funding institutions, especially NIH and NSF, should offer human subjects training specific to CBPR research, and should sensitize universities to the importance of supporting community groups. University IRBs should be aware that community organizations may operate on different timelines, and that the intense and lengthy university IRB reporting process can create conflicts for them. Even when giving this guidance to IRBs, researchers doing grant-funded community-based research would be wise to include ample time for IRB review in their grant proposals.

Funding agencies should encourage academic institutions to provide IRB oversight to both academic and community partners to avoid unnecessary delays and expenses in protocol reviews. They may want to promote consortium-based approval whereby one institution's IRB is accepted by others in the consortium (Silent Spring Institute has had this experience with Boston University, a partner in some of its other projects; indemnification may be necessary so that universities do not bear responsibility for the actions of community partners). Funding agencies should ensure that all community partners in the projects they sponsor will have the resources necessary to complete the IRB process and comply with reporting requirements. In addition, funding agencies could help communicate to IRBs that community groups should not have to expend their own resources to navigate the review process. Funding institutions could use grant announcements and descriptions to give clear guidance about the IRB issues that CBPR partners are likely to face (another possible avenue for advocacy work is the Applied Research Ethics National Association, an organization that provides resources and information about ethical and procedural issues of campus IRBs [25]).

#### 4) Regulate any conflicts of interest IRBs may bring to the review process

For example, Massachusetts DPH RaDAR Committee has a role to play in protecting the confidentiality of data obtained from the state cancer registry, but its role in human subjects reviews must be circumscribed to avoid conflicts of interest when the agency might have a vested interest in the outcome of a proposed study (because of its implications for public health agency action, for example). We should note that the statutory regulations governing IRBs state that "The IRB should not consider possible long-range effects of applying knowledge gained in the research (for example, the possible effects of the research on public policy) as among those research risks that fall within the purview of its responsibility." (45 CFR 46.111(a)(2)).

Creating an independent IRB for such situations would protect human subjects while avoiding conflicts of interest that could hinder the progress of a worthy study. That IRBs can have their own conflict of interest is a legitimate concern. A recent survey of 893 IRB members at 100 academic institutions found that 36 percent of IRB members had at least one relationship with industry in the previous year, of which only two-thirds had been disclosed to the IRB. Of those reporting conflicts, nearly one-third had participated in the reviews anyway [26]. Of course, some have argued that conflicts of interest are widespread even among independent IRBs [27], so further action may be needed.

#### 5) Reassess how IRBs oversee situations in which participants desire access to and disclosure of their own study results

In some cases, this necessitates continued interaction between researchers and participants, a process that IRBs may be reluctant to allow and are poorly-designed to manage. Iterative rounds of approval for ongoing communication with study participants will result in delays that undermine researchers' relationships with participants, and harms participants' capacity to take action to reduce their exposures. Participants may also want to share their personal results with other study participants and have the collective power to disseminate their results through their own networks and broader public forums. Putting the brakes on individual report-back could push confidentiality protections to collide with the principle of beneficence. Thus, CBPR challenges IRBs to reassess the seemingly contradictory elements of the Belmont principles, and develop alternatives that do not require choosing one principle over another. Seen another way, IRB perspectives on confidentiality may in fact be contrary to federal law, as we noted earlier in our point on (45 CFR 46.111(a)(7), which approaches confidentiality "when appropriate." As with the earlier regulatory points on competence to review CBPR proposals and regarding concern for public policy effects, this is one of several issues in which IRBs may not be properly heeding existing regulatory requirements.

#### 6) Encourage CBPR researchers and partners to educate IRBs about flexibility in regulations

As pointed out in two above sections, IRBs are not always aware of the flexibility in federal regulations about confidentiality and about competency to conduct community-oriented reviews. We believe that CBPR projects can make it easier for IRBs to support them if they remind IRBs about such flexibility.

### Working with community and tribal Institutional review boards

Academic IRBs are not the only forum in which community benefits of the research may be assessed. Some communities have convened their own review boards to assess collectively whether proposed research is justified and benefits the community [28]. For example, the Navajo Nation maintains its own IRB to protect its people from research that would not directly help them [29]. The Indian Health Service adds "respect for *communities*" to the Belmont principles and expects proposals to discuss whether there are tribal consultants who could be involved, whether there are community capacity-building benefits to the tribe, whether researchers have an understanding of community research priorities, and whether researchers will provide regular and timely community consultations. Similarly, the citizens' organization that oversees research on residential exposures from the Fernald, Ohio, nuclear weapons plant permits researchers access to its records only when there is a concrete benefit for the community, regardless of academic IRB approval [30].

Increasing numbers of researchers involved in community-based participatory research seek community review boards that emphasize community protections alongside traditional IRB responsibilities [31]. While tribal IRBs have the power of regular IRBs, other community review boards do not yet meet the requirements for oversight of federally funded research, requiring an additional IRB to provide formal guarantees. Although an increasing number of communities now oversee research, factors such as geographic dispersion, political disorganization, and the lack of authority to review research protocols prevent other communities from doing the same. Academic IRBs reviewing research proposals on behalf of such communities need to understand their form and organization, their communal needs and vulnerabilities, and their existing governance and communication structures for disseminating research [32].

Community representation in the review process would be helpful not only to those explicitly engaged in community research but also to those engaged in individual research who may not have considered the effects of their research on communities. NIH rules were clarified in 1998 to ensure that IRBs have "knowledge of the local research context," but while one member of the IRB must be from outside the institution, direct community representation is not required [33]. Community representation on academic IRBs usually takes the form of large, well-established organizations rather than grassroots groups, and does not usually reflect the demographic composition of the communities under study [34]. While two 2001 reports from the Office of Human Subjects Research of NIH and the National Bioethics Advisory Commission delineate the need to expand community involvement in research beyond mere representation on IRBs [35], most pressure for deep community involvement stems from activist groups [25]. We recommend that IRBs recruit not just any community members, but those that have experience in either CBPR or other community-engaged research. This can provide benefits to many IRB reviews, not only CBPR ones, because of the creativity, flexibility, and respect for human subjects protection that comes with CBPR experience.

### Limitations of this study and recommendations for future research

Our understanding of IRB's lack of familiarity with CBPR principles comes from our knowledge of a small number of IRBs dealing directly with CBPR researchers. We did not conduct a survey that would be representative of all IRBs, and hence our findings are not necessarily generalizable. However, we do think that the IRBs dealing with CBPR scholars would be more likely than other IRBs to have such familiarity, based on past applications by researchers to those IRBs. To better understand how IRBs in general deal with CBPR principles and the right-to-know that we emphasize, we recommend broad surveys of IRBs.

We recommend that health and social science researchers study a large sample of scientists working in human exposure and related areas to gather more detailed information on IRB challenges. It is important to learn whether and how IRBs may have changed their procedures in response to pressure for individual report-back and community-level protection, and whether and how researchers may have changed their beliefs about or methods for the reporting of individual data. Extensive interviews with IRB staff and academic members should supplement interviews with researchers. Future research should study how participants experience the process of informed consent in biomonitoring and household exposure studies, the degree to which they desire detailed information and supplementary information used for reducing exposure, and the ways they use their personal data to explain exposure, understand disease causation, and to seek regulatory and other changes.

## Conclusions

CBPR researchers report that IRBs are not generally attuned to their particular needs, due to their emphasis on individual consent that is based on a clinical model and their lack of understanding about the importance of community-level consent and the need to share individual data with participants. In short, the very CBPR practices that concern many IRBs are exactly those that make community-engaged work so valuable for researching and addressing environmental justice issues. Resolving this tension requires reforming the human subjects review process in a way that improves CBPR rather than hinders it. Further, some of the IRB restrictions on common CBPR practices, such as sharing results with individual study participants, may actually violate federal regulations on human subjects protection and counter the ethical concerns of study communities. Efforts to overcome these IRB challenges require a more holistic understanding of how CBPR researchers and study communities (whether defined by geography, class, ethnicity, or other socially salient distinctions) collaborate in ways that empower community organizations to play a central role in the research process, which includes human subjects protection and ethical oversight. Ultimately, IRBs will need to go beyond simply modifying traditional oversight procedures to fundamentally incorporating how CBPR ethics redefines the research enterprise itself, including researcher-participant relationships, academic-community interaction, and the right-to-know about chemicals in people's environments, homes, and bodies.

## Appendix 1: Strategies for Advancing Multi-Partner CBPR Projects Through the IRB Process

### Strategies for Researchers and Community Partners

• Educate IRB staff and board members about the objectives and research methods of CBPR

• Make sure academic IRBs know community partners

• Share positive models and problematic experiences with other teams, IRBs, and funding institutions

### Strategies for IRBs and Funding Institutions

• Keep abreast of CBPR and other cutting-edge research approaches

• Develop routine procedures for the review of CBPR projects

• Provide guidance and tools for navigating IRB issues unique to academic-community collaboratives

• Regulate any conflicts of interest IRBs may bring to the review process

• Reassess how IRBs oversee situations in which participants desire access to and disclosure of their own study results

• Encourage CBPR researchers and partners to educate IRBs about flexibility in regulations

## Abbreviations

CBE: Communities for a Better Environment; CBO: community-based organization; CBPR: community-based participatory research; CITI: Collaborative Institutional Training Initiative; DPH: Department of Public Health; EDCs: endocrine disrupting compounds; IRB: Institutional Review Board; NIEHS: National Institute of Environmental Health Sciences; PBDEs: polybrominated diphenyl ethers; RaDAR: Research and Data Access Review

## Competing interests

The authors declare that they have no competing interests.

## Authors' contributions

PB conceived the study, conducted some of the interviews, made the conference presentation that led to the article, and led the article writing. RMF conducted some of the interviews and played a major role in writing and editing the article. JB led the larger study from which this study arose, conducted some of the interviews, assisted in the conference presentation, and worked on writing the article. RGA conducted some of the interviews, assisted in the conference presentation, and worked on writing the article. RAR played a major role in the conference presentation and assisted with the article writing. LS researched the background literature and assisted in writing the article. CP provided data about the community partners' roles in dealing with the IRB, and assisted in the conference presentation. RS played assisted in editing and writing the final version of the article for submission.
